# Effect of adding citrus flavonoid (Bioflavex) to diet on growth, feed efficiency, rumen histomorphology, carcass traits and meat quality of lambs

**DOI:** 10.3389/fvets.2025.1572911

**Published:** 2025-04-02

**Authors:** Saleh Al-Ghamdi, Hani H. Al-Baadani, Abdulrahman S. Alharthi, Gamaleldin M. Suliman, Ibrahim A. Alhidary

**Affiliations:** ^1^Department of Agricultural Engineering, College of Food and Agriculture Science, King Saud University, Riyadh, Saudi Arabia; ^2^Department of Animal Production, College of Food and Agriculture Science, King Saud University, Riyadh, Saudi Arabia

**Keywords:** lamb, flavonoid, performance, carcass, meat quality

## Abstract

Citrus flavonoids (Bioflavex) are plant polyphenols with antioxidant properties that can have a positive effect on growth, rumen health, carcass characteristics and meat quality in ruminants. In this study, the effects of adding citrus flavonoids to the diet on growth performance, feed efficiency, rumen morphology, carcass characteristics, and meat quality of Awassi lambs were investigated. Thirty-six male lambs (27.36 ± 0.025 kg initial body weight) at 14 weeks of age were individually allocated to 3 dietary treatments (T1 = basal diet without any additives, T2 = basal diet with 0.4 g Bioflavex/kg diet dry matter and T3 = basal diet with 0.8 g Bioflavex/kg diet dry matter) with 12 lambs as replicates per treatment in a completely randomized design. Performance was evaluated, including body weight, weight gain, growth rate and feed conversion ratio over 56 days. At the end of the study, all lambs were slaughtered to measure rumen histomorphology and carcass and meat characteristics. The results showed that T2 and T3 had higher growth indicators, carcass weights of hot and cold and better feed conversion than T1 (*p* < 0.05). In addition, lambs fed T2 and T3 had higher rumen histomorphology parameters (papilla length, papilla width, papilla surface area, and total surface of papillae) than lambs fed T1 (*p* < 0.05). Shoulder weight, backfat thickness, body wall fat and carcass redness decreased, while foreshank and breast weight increased with the addition of Bioflavex (*p* < 0.05). Shear force, cooking loss, water holding capacity, and myofibril fragmentation index were lower with Bioflavex than with T1 (*p* < 0.05). In conclusion, the study showed that supplementation with citrus flavonoids (0.8 g Bioflavex/kg diet dry matter) can have a positive effect on lamb growth, rumen development and meat quality.

## Highlights

- Growth indicators increased and feed conversion ratio improved by Bioflavex.
- The size and surface area of the rumen papillae increased in the treated lambs.
- The addition of Bioflavex to the diet of lambs led to a higher carcass weight with a simultaneous reduction in the thickness of back fat and body wall fat.
- Bioflavex improved the tenderness of the meat and reduced cooking losses.

## 1 Introduction

Lamb production plays an important role in the agricultural economy of many countries, and improving growth performance and meat quality remains a major challenge (1, 2). Various factors, including feed composition, additives, genetics, and environmental conditions, significantly influence these traits (3). In addition, there has been an increasing trend in recent years toward the use of natural feed additives to enhance lamb performance and improve meat quality while reducing the use of antibiotics as growth promoters (4). Feed additives for lambs can influence various aspects of animal performance by improving feed efficiency, resulting in higher weight gain and better feed conversion ratio (5). Feed additives, especially those with antimicrobial or antioxidant properties, can influence the microbial environment in the rumen, improve rumen fermentation and increase nutrient utilization (6). Feed additives can influence carcass composition by increasing lean meat production, reducing fat deposition and improving meat quality traits such as tenderness, juiciness, flavor and color (7).

Citrus flavonoids (bioflavex) are plant polyphenols with multiple biological activities (8). In particular, citrus flavonoids have been shown to have antioxidant, anti-inflammatory and antimicrobial properties in ruminants (9). Bioflavex, a standardized extract of citrus flavonoids, has attracted attention for its potential to improve animal health and productivity (10). Several studies have shown that various flavonoids have a positive effect on growth performance, carcass characteristics and meat quality in lambs. For example, Paniagua et al. (11) reported that the addition of flavonoids to feed improved feed efficiency and carcass yield in lambs. In another study by Simitzis et al. (12) it was observed that the addition of flavonoids to the feed improved the antioxidant status and meat quality of lambs. In addition, the addition of citrus flavonoids to the diet improved feed efficiency, carcass characteristics and rumen histomorphology (9, 13). The addition of citrus flavonoid extract to the diet proved to be a possible alternative to antibiotics in cattle (4).

However, research on the specific effects of Bioflavex on lamb performance and meat quality is limited. The main hypothesis of this study is that the inclusion of Bioflavex in the diet of growing lambs increases daily weight gain, improves feed efficiency, and improves rumen morphology and carcass and meat characteristics. Therefore, the present study aims to investigate the effects of adding citrus flavonoids to the diet of growing Awassi lambs. Specifically, the study investigates the effects of Bioflavex on growth performance, feed efficiency, rumen morphology, carcass characteristics, and meat quality.

## 2 Materials and methods

### 2.1 Ethical considerations

This study, including the study design, animals used, and sampling, complied with all guidelines of the King Saud University Scientific Research Ethics Committee (KSU-SE−22-26).

### 2.2 Lambs and design

A total of thirty-six newly weaned male Awassi lambs (27.36 ± 0.025 kg) aged 14 weeks obtained from a local company (Al-Khalidiyah Co., Riyadh, Saudi Arabia) were randomly allocated into individual pens (2 m^2^) for each of the three dietary treatments with 12 lambs (one lamb per pen) for each dietary treatment, each lamb representing a replicate (experimental unit) using a completely randomized design. The sample size of 12 lambs per dietary treatment was chosen to provide sufficient statistical power. The basal diet was formulated as a complete pellet diet that met all the nutritional requirements of the lambs during the growing period, as recommended by the National Research Council (14) as shown in the Table 1. The dietary treatments were as follows: T1 = lambs received the basal diet without any additives, T2 = lambs received the basal diet with an addition of 0.4 g Bioflavex/kg diet dry matter and T3 = lambs received the basal diet with an addition of 0.8 g Bioflavex/kg diet dry matter. The commercially available Bioflavex® (Interquim SA, Sant Cugat, Barcelona, Spain) is citrus flavonoid extract consisting mainly of naringin extracted from bitter oranges (*Citrus aurantium*) and grapefruits (*Citrus paradisi*). It also contains neohesperidin, poncirin, and traces of hesperidin, isonaringin, and neoeriocitrin. The selection of 0.4 and 0.8 g Bioflavex/kg diet dry matter were based on the manufacturer’s recommendations and reference levels from previous studies on similar flavonoid sources in Holstein bulls (11, 13). Water and feed were provided ad libitum, with adequate ventilation and hygiene ensured throughout the 56-day experiment. Prior to the start of the experiment, all health and prevention guidelines were followed on arrival of the animals and they were gradually acclimatized to pelleted complete feed for 10 days. During this period, the lambs were vaccinated against the usual diseases (internal and external parasites, peste des petits ruminants, and septicemia).

**Table 1:** Feedstuffs and nutrient composition of basal diet of growing Awassi lambs.

Items	Content (%)
Yellow corn	30.00
Barley	17.60
Wheat bran	5.20
Palm kernel meal	7.45
Soybean hulls	10.55
Wheat straw	15.00
Alfalfa hay	6.30
Sodium chloride	0.47
Calcium carbonate	2.58
Molasses	4.60
Commercial premix^*^	0.25
Total	100.00
Nutrients analysis	%
Dry matter	92.43
Crude protein	12.87
Crude fiber	11.98
Ether extract	5.33
Neutral detergent fiber	32.57
Acid detergent fiber	17.76
Ash	8.01
	Kcal/kg
Metabolizable energy	2,830

*The premix composition per kilogram included 13,000 IU vitamin A, 1,300 IU vitamin D, 25 IU vitamin E, 400 mg magnesium, 70 mg zinc, 30 mg copper, 85 mg manganese, 0.40 mg selenium, and 0.80 mg cobalt.

The basal diet was analyzed in triplicate to determine the nutrient composition. Dry matter was determined by drying in an oven at 105°C for 24 h. Crude protein was measured using the Kjeldahl method, multiplying the nitrogen content by 6.25. The ether extract was determined using the Soxhlet method. Crude fiber and ash were analyzed according to the standard methods of the Association of Official Analytical Chemists (15). Neutral detergent fiber and acid detergent fiber were analyzed using a Fiber Analyzer (ANKOM Technology, New York, NY, United States) according to the Van Soest method (16). The metabolizable energy was estimated by multiplying the digestible energy (gross energy after considering the energy losses of the diet during digestion and absorption) by the general correction factor 0.82 (17).

### 2.3 Growth and feed efficiency measures

Growth and feed efficiency were assessed in three phases: early (1–28 days), mid (29–56 days), and overall (1–56 days). At the initial and final of each phase, body weights of individual lambs were recorded using a calibrated digital scale to calculate daily weight gain and relative growth rate according to previously described methods (18). Daily weight gain was calculated as (final body weight - initial body weight) / number of days. The RGR was determined using the following formula: Relative growth rate = [2 * (final body weight- initial body weight)] / (initial body weight + final body weight) * 100. Daily feed intake was determined by accurately weighing the feed offered at the beginning of each phase and subtracting the amount remaining at the end. Daily feed intake = (feed offered - feed remaining) / number of days (19). Feed conversion ratio, an indicator of feed efficiency, was calculated as follows: Feed conversion ratio = daily feed intake / daily weight gain (20).

### 2.4 Slaughter procedures

At the end of the experimental period (56 days), all lambs (12 lambs per dietary treatment) were fasted overnight (approximately 12 h) and had free access to water. The lambs were then transported to the Diriyah slaughterhouse in Riyadh, Kingdom of Saudi Arabia, in a specially designed transport vehicle. The lambs were then humanely slaughtered at this abattoir under strict veterinary supervision and in compliance with all relevant animal welfare guidelines. The weight before slaughter was recorded individually.

### 2.5 Histomorphology measurement of the rumen

Immediately after slaughter, a representative sample of rumen tissue (about 2 cm) was collected in triplicate from the central region of the ventral rumen sac (midway between ventral coronary pillar and cranial pillar) of each lamb after slaughter. The samples were fixed in 10% neutral buffered formalin and then histologically processed according to Petrič et al. (21). The fixed rumen tissue samples were processed for histological analysis using standard procedures, including dehydration in graded alcohols, clarification in xylene, and embedding in paraffin (Tissue-Tek VIP 5 Jr., Sakura, Japan). 5 micrometer- thick sections were cut with a microtome (Leica Bio-systems, Germany) and stained with hematoxylin and eosin (H and E) to perform morphometric measurements (height, width, and density of the rumen papillae) using a digital imaging system and image analysis software (Nikon, Corp, Japan). The papilla surface area and total surface area were calculated according to the previously described methods by Sohail et al. (22).

### 2.6 Carcass characteristics

After slaughter, all lambs were skinned individually and weight was measured immediately after slaughter (hot carcass weight) and again after 24 h chilling at 4°C (cold carcass weight). Cold shrink was calculated as (hot carcass weight - cold carcass weight) / hot carcass weight * 100, according to the previously described methods (23). Dressing percentage was calculated using the following formula: Dressing = hot carcass weight / slaughter weight * 100 (24). The carcass compactness index (Kg/cm) was calculated as (cold carcass weight /internal carcass length) according to Carvalho et al. (25). The weights of various organs (head, heart, lungs, liver, spleen, kidneys, tail, skin, feet, rumen and intestine) were recorded. The organ weights were expressed as a percentage of the slaughter weight (26).

### 2.7 Carcass cutting and fat deposition

The carcasses of 12 lambs per dietary treatment were split longitudinally into two halves. Each half was cut into the most important parts: shoulder, rack, loin, leg, foreshank and breast. The weight of each cut was recorded and expressed as a percentage of the weight of the half carcass (27). The thickness of subcutaneous fat was measured at two locations: above the longissimus thoracis muscle (back fat) and along the body wall (24). The weights of the main internal fat depots (kidney knob fat, pericardial fat, and omental fat) were determined and expressed as a percentage of slaughter weight (26).

### 2.8 Physicochemical properties

The physicochemical properties of the meat (longissimus dorsi muscle) and the carcass wall for each lamb carcass (12 lambs per dietary treatment) were evaluated in triplicate at two time points: immediately after slaughter (initial) and after 24 h of chilling (ultimate). The color of the meat and carcass (lightness, redness and yellowness) was measured using a Konica Minolta CR − 400 colorimeter (28). The pH of the meat was determined using a Hanna 211 pH meter equipped with a piercing electrode and an integrated thermometer (28).

### 2.9 Meat characteristics

Twelve longissimus dorsi muscle samples (one per lamb) were taken from each treatment group. Each sample was divided into five portions in triplicate and vacuum-packed for storage at −20°C. Cooking loss was determined by weighing the samples before and after cooking on an electric grill (Princess 2,321, Netherlands) at an internal meat temperature of 70°C. The cooking loss (%) was calculated as: [(initial weight - final weight)/initial weight] x 100 (24). Water holding capacity was measured using a press method (12 kg) with filter paper for 5 min, as previously described (29). Water holding capacity was calculated as the percentage of water retained in the meat. The myofibril fragmentation index was determined to assess the degradation of muscle protein (30). Meat samples were homogenized and absorbance at 540 nm was measured using a Jenway 6,705 spectrometer. Higher absorbance values indicate greater fragmentation of myofibrils and potentially greater tenderness. Shear force and texture pattern parameters (hardness, elasticity, cohesion, chewiness) were measured using a TA-HD texture analyzer with a Warner-Bratzler blade (30).

### 2.10 Statistical data analysis

A statistical power analysis was performed with G*Power 3.1. Based on the observed Cohen’s d effect sizes and the sample size (*n* = 12 lambs per dietary treatment), the power to detect significant differences at *α* = 0.05 was between 0.72 and 0.91, indicating that the study had sufficient power to detect the observed effects. Normality of the data was assessed using skewness and kurtosis tests and visual inspection of box plots. To assess the effects of the three dietary treatments (T1-T3), a one-way analysis of variance (ANOVA) was performed using a general linear model (GLM) within SAS 9.4 statistical software (31). The model was formulated as follows: Observation (Yij) = overall mean (*μ*) + treatment effect (i) + experimental error (eij). Significant differences between dietary treatments were determined using Tukey’s Honestly Significant Difference (HSD) test at a significance level of *p* < 0.05. Data are presented as means ± standard error of the mean (SEM).

## 3 Results

### 3.1 Growth and feed efficiency results

Table 2 shows the effects of adding Bioflavex to the pelleted complete diet on the growth and feed efficiency of Awassi lambs. In the early phase (1–28 days), there was no significant difference (*p* > 0.05) in body weight between the dietary treatments. In addition, the results showed that lambs fed 0.4 and 0.8 g Bioflavex per kg diet dry matter (T2 and T3) had higher body weight, daily weight gain and relative growth rate and better feed conversion ratio compared to lambs fed basal diet (T1; *p* < 0.05) in early, mid, and overall phases (1–28 days, 29–56 days, and 1–56 days). On the other hand, adding Bioflavex to diets had not affected daily feed intake than in lambs fed T1 (*p* > 0.05).

**Table 2:** The effects of adding Bioflavex to pelleted complete diet on the growth and feed efficiency of Awassi lambs.

Items	Dietary treatments^1^			SEM^2^	*p*- value
	T1	T2	T3		
From 1–28 days					
Body weight (kg) at 1 day	27.37	27.38	27.34	0.02	0.995
Body weight (kg) at 28 days	32.19^b^	34.12^a^	34.81^a^	1.05	0.027
Daily weight gain (g)	176^b^	241^a^	270^a^	9.94	<0.001
Relative growth rate (%)	16.76^b^	22.20^a^	24.54^a^	1.10	<0.001
Daily feed intake (g)	1,341	1,351	1,381	31.55	0.652
Feed conversion ratio (g/g)	7.74^a^	5.69^b^	5.18^b^	0.29	<0.001
From 29–56 days					
Body weight (kg) at 56 days	38.08^b^	41.09^a^	42.68 ^a^	0.98	0.010
Daily weight gain (g)	210^c^	249^b^	281^a^	9.28	0.011
Relative growth rate (%)	16.92^b^	18.85^a^	20.54^a^	0.93	0.026
Daily feed intake (g)	1,436	1,454	1,552	56.98	0.314
Feed conversion ratio (g/g)	6.95^a^	5.99^b^	5.70^b^	0.39	0.045
From 1–56 days					
Daily weight gain (g)	193^b^	245^a^	276^a^	9.73	<0.001
Relative growth rate (%)	33.42^c^	40.58^b^	44.48^a^	2.32	0.008
Daily feed intake (g)	1,388	1,402	1,466	39.88	0.349
Feed conversion ratio (g/g)	7.26^a^	5.78^b^	5.40^b^	0.25	<0.001

Significant differences (*p* < 0.05) between dietary treatments are indicated by superscript letters (a–c) in each row. ^1^T1 = lambs fed the basal diet without any supplements, T2 = supplemented with 0.4 g Bioflavex/kg diet dry matter, and T3 = supplemented with 0.8 g Bioflavex/kg diet dry matter. ^2^SEM is standard error of means.

### 3.2 Rumen histomorphology

Table 3 shows the effects of adding Bioflavex to the pelleted complete diet on the rumen histomorphology of Awassi lambs. The results show that lambs fed 0.4 and 0.8 g Bioflavex per kg diet dry matter (T2 and T3) had higher rumen histomorphology parameters, including papilla length, papilla width, papilla surface area, and total surface of papillae than lambs fed T1 (*p* < 0.05). In addition, lambs fed T3 had higher papilla length, papilla width, and total surface of papillae compared to lambs fed T2. On the other hand, adding Bioflavex to diets had not affected papilla density of rumen compared to in lambs fed T1 (*p* > 0.05).

**Table 3:** The effects of adding Bioflavex to pelleted complete diet on the rumen histomorphology of Awassi lambs.

Items	Dietary treatments^1^			SEM^2^	*p*- value
	T1	T2	T3		
Papilla length (mm)	1.65^c^	1.88^b^	1.96^a^	0.07	0.009
Papilla width (mm)	0.24^c^	0.26^b^	0.29^a^	0.01	0.006
Papilla surface area (mm^2^)	1.22^b^	1.53^a^	1.76^a^	0.08	0.002
Density (n/cm^2^)	87	99	109	7.58	0.145
Total surface of papillae (mm^2^/cm^2^)	69.65^c^	97.46^b^	120.51^a^	4.29	0.001

Significant differences (*p* < 0.05) between dietary treatments are indicated by superscript letters (a-c) in each row. ^1^T1 = lambs fed the basal diet without any supplements, T2 = supplemented with 0.4 g Bioflavex/kg diet dry matter, and T3 = supplemented with 0.8 g Bioflavex/kg diet dry matter. ^2^SEM is standard error of means.

### 3.3 Characteristics of the carcass and body organs

Table 4 shows the effects of adding Bioflavex to the pelleted complete diet on characteristics of the carcass and body organs of Awassi lambs. The results showed that the absolute weights of the slaughter, hot carcass, and cold carcass were higher in lambs fed 0.4 and 0.8 g Bioflavex per kg diet dry matter (T2 and T3) than in lambs fed T1 (*p* < 0.05). Whereas, adding Bioflavex to diets had not affected cold shrink, dressing, internal carcass length, and carcass compactness index than in lambs fed T1 (*p* > 0.05). The relative weights of the various body organs also did not differ significantly between dietary treatments (*p* > 0.05).

**Table 4:** The effects of adding Bioflavex to pelleted complete diet on the carcass characteristics of Awassi lambs.

Items	Dietary treatments^1^			SEM^2^	*p*- value
	T1	T2	T3		
Carcass traits					
Slaughter weight (kg)	38.22^b^	41.04^a^	42.74^a^	0.99	0.010
Hot carcass weight (kg)	18.43^b^	20.57^a^	21.34^a^	0.73	0.041
Cold carcass weight (kg)	18.14^b^	20.27^a^	21.07^a^	0.73	0.039
Cold shrink (%)	1.57	1.44	1.28	0.15	0.471
Dressing (%)	48.24	50.12	49.98	0.67	0.132
Internal carcass length (cm)	64.4	65.2	66.4	1.69	0.710
Carcass Compactness Index (kg/cm)	0.28	0.30	0.30	0.01	0.646
Relative weight of various organs (%)					
Head (Skinned)	3.20	3.18	3.11	0.09	0.808
Heart	0.36	0.40	0.40	0.01	0.153
Lungs	1.26	1.06	1.00	0.08	0.134
Liver	1.57	1.37	1.31	0.08	0.102
Spleen	0.16	0.16	0.14	0.01	0.260
Kidneys	0.25	0.27	0.24	0.02	0.763
Tail	5.38	5.07	4.50	0.38	0.289
Skin	12.33	12.77	12.57	0.69	0.904
Feet	2.57	2.45	2.66	0.10	0.426
Rumen	9.77	9.57	10.63	0.64	0.489
Intestine	6.17	6.43	6.57	0.20	0.406

Significant differences (p < 0.05) between dietary treatments are indicated by superscript letters (a, b) in each row. ^1^T1 = lambs fed the basal diet without any supplements, T2 = supplemented with 0.4 g Bioflavex/kg diet dry matter, and T3 = supplemented with 0.8 g Bioflavex/kg diet dry matter. ^2^SEM is standard error of means.

### 3.4 Carcass cutting and fat deposition

Table 5 shows the effects of adding Bioflavex to the pelleted complete diet on carcass cutting and fat deposition of Awassi lambs. There was no significant difference (p > 0.05) in half carcass weight between the dietary treatments. On the other hand, the results showed that lambs fed T3, followed by T2 had lower relative shoulder weights compared to lambs fed T1 (*p* < 0.05). Lambs fed T2 and T3 had higher foreshank and breast weight compared to lambs fed T1 (*p* < 0.05). In addition, lambs fed T3 (0.8 g Bioflavex per kg diet dry matter) had lower relative rack weight and higher loin weight than lambs fed T1 and T2 (*p* < 0.05). Lambs fed T3 had higher relative leg weight than lambs fed T1 and T2, whereas T2 had lower than lambs fed T1 (*p* < 0.05). The relative weights of fat deposition including kidney knob fat, pericardial fat and omental fat were not affected by dietary treatments (*p* > 0.05). The thickness of backfat and body wall fat were lower in lambs fed 0.4 and 0.8 g Bioflavex per kg diet dry matter (T2 and T3) than in lambs fed T1 (*p* < 0.05).

**Table 5:** The effects of adding Bioflavex to pelleted complete diet on the carcass cutting and fat deposition of Awassi lambs.

Items	Dietary treatments^1^			SEM^2^	*p*- value
	T1	T2	T3		
Carcass cutting					
Half carcass weight (kg)	7.57	7.69	8.29	0.33	0.309
Shoulder (%)	28.17^a^	25.89^b^	23.77^c^	0.33	<0.001
Rack (%)	10.36^a^	10.69^a^	9.27^b^	0.17	<0.001
Loin (%)	14.26^b^	13.92^b^	15.37^a^	0.21	0.001
Leg (%)	32.57^b^	30.35^c^	34.15^a^	0.71	0.009
Foreshank and breast (%)	14.64^b^	19.15^a^	17.44^a^	0.54	<0.001
Fat deposition					
Kidney knob fat (%)	1.60	1.19	1.44	0.11	0.073
Pericardial fat (%)	0.12	0.15	0.16	0.01	0.070
Omental fat (%)	2.09	1.70	1.93	0.11	0.081
Backfat (mm)	6.60^a^	4.60^b^	4.10^b^	0.20	<0.001
Body Wall Fat (mm)	10.80^a^	8.50^b^	8.40^b^	0.37	0.001

Significant differences (p < 0.05) between dietary treatments are indicated by superscript letters (a–c) in each row. ^1^T1 = lambs fed the basal diet without any supplements, T2 = supplemented with 0.4 g Bioflavex/kg diet dry matter, and T3 = supplemented with 0.8 g Bioflavex/kg diet dry matter. ^2^SEM is standard error of means.

### 3.5 Physicochemical properties

Table 6 shows the effects of adding Bioflavex to the pelleted complete diet on the physicochemical properties of the Awassi lambs. The results for meat pH did not differ significantly between dietary treatments (*p* > 0.05). Meat and carcass color (lightness, redness, and yellowness) were also not affected by the different dietary treatments (*p* > 0.05), except for the initial and ultimate carcass wall redness color score, which was lower in lambs receiving 0.4 and 0.8 g Bioflavex per kg diet dry matter (T2 and T3) than in lambs receiving T1 (*p* < 0.05). In addition, lambs fed T2 had lower initial and ultimate carcass wall redness color score compared to lambs fed T3.

**Table 6:** The effects of adding Bioflavex to pelleted complete diet on the physicochemical properties of Awassi lambs.

Items	Dietary treatments^1^			SEM^2^	*p*- value
	T1	T2	T3		
Initial meat color and pH values					
pH	5.79	5.84	5.78	0.07	0.864
Lightness	40.25	38.84	38.59	2.15	0.842
Redness	14.06	13.16	14.70	0.76	0.389
Yellowness	7.27	6.62	7.96	0.67	0.405
Ultimate meat color and pH values					
pH	5.57	5.56	5.58	0.03	0.924
Lightness	41.10	42.89	43.22	1.76	0.665
Redness	14.20	16.31	15.50	0.82	0.232
Yellowness	14.02	13.99	12.12	0.90	0.275
Initial carcass color values					
Lightness	73.15	78.02	75.74	1.44	0.097
Redness	-2.47^a^	-4.32^c^	-3.71^b^	0.37	0.013
Yellowness	8.80	7.61	8.37	0.48	0.256
Ultimate carcass color values					
Lightness	72.04	75.04	74.70	1.56	0.366
Redness	1.74^a^	-0.67^c^	1.08^b^	0.61	0.042
Yellowness	14.42	14.17	15.13	0.53	0.447

Significant differences (*p* < 0.05) between dietary treatments are indicated by superscript letters (a–c) in each row. ^1^T1 = lambs fed the basal diet without any supplements, T2 = supplemented with 0.4 g Bioflavex/kg diet dry matter, and T3 = supplemented with 0.8 g Bioflavex/kg diet dry matter. ^2^SEM is standard error of means.

### 3.6 Meat characteristics

Table 7 shows the effects of adding Bioflavex to the pelleted complete diet on the meat characteristics of Awassi lambs. The results showed that lambs fed 0.4 and 0.8 g Bioflavex per kg diet dry matter (T2 and T3) had lower shear force, cooking loss, water holding capacity, and myofibril fragmentation index compared to lambs fed T1 (*p* < 0.05). In addition, lambs fed T3 had lower shear force, cooking loss, and myofibril fragmentation index compared to lambs fed T2. There was no significant difference (*p* > 0.05) in terms of hardness, springiness, and cohesiveness between the dietary treatments. On the other hand, lambs fed T2 (0.4 g Bioflavex per kg diet dry matter) had higher chewiness than lambs fed T1 and T3 (*p* < 0.05).

**Table 7:** The effects of adding Bioflavex to pelleted complete diet on the meat characteristics of Awassi lambs.

Items	Dietary treatments^1^			SEM^2^	*p*- value
	T1	T2	T3		
Shear force (N/cm^2^)	7.91^a^	6.34^b^	5.20^c^	0.35	<0.001
Cooking loss (%)	42.89^a^	41.12^b^	39.59^c^	0.39	<0.001
Water-holding capacity (%)	0.38^a^	0.34^b^	0.34^b^	0.01	<0.001
Myofibril fragmentation index	52.94^a^	43.85^b^	34.84^c^	1.06	<0.001
Texture profile analysis					
Hardness (N)	4.20	3.87	3.46	0.19	0.064
Springiness	0.77	0.82	0.84	0.02	0.123
Cohesiveness	0.62	0.66	0.67	0.01	0.185
Chewiness	2.12^b^	2.80^a^	1.88^b^	0.14	0.002

Significant differences (*p* < 0.05) between dietary treatments are indicated by superscript letters (a–c) in each row. ^1^T1 = lambs fed the basal diet without any supplements, T2 = supplemented with 0.4 g Bioflavex/kg diet dry matter, and T3 = supplemented with 0.8 g Bioflavex/kg diet dry matter. ^2^SEM is standard error of means.

## 4 Discussion

Citrus flavonoids (Bioflavex) are a feed additive for ruminants that has been shown to have a multi-faceted mechanism of action, contributing to improved production performance, rumen health, carcass characteristics and meat quality (10). Due to their phenolic structure, citrus flavonoids have strong antioxidant properties that reduce oxidative stress in the rumen by reducing damage to rumen epithelial cells and thus improving overall rumen function (9). A study by Paniagua et al. (13) reported that the inclusion of citrus flavonoids in the diet of cattle can alter the rumen ecosystem by stimulating the growth of beneficial bacteria, such as cellulolytic bacteria, which are important for fiber digestion and volatile fatty acid production, leading to improved nutrient utilization. Some studies have shown that flavonoids can improve meat quality such as tenderness, juiciness and color stability by influencing factors such as oxidative stability and protein degradation (12).

Lambs fed 0.4 and 0.8 g Bioflavex per kg diet dry matter had significantly higher body weight, daily weight gain and RGR and improved feed conversion ratio throughout the study period. Interestingly, the study found no significant difference in daily feed intake between the dietary treatments. This suggests that the improved growth performance with the addition of Bioflavex is not due to increased daily feed intake, but to improved nutrient utilization and metabolic efficiency. Some studies have shown that flavonoids can stimulate the release of growth hormones, which can promote growth (2). A previous study hypothesized that Bioflavex may have altered the composition and activity of the rumen microbiota, resulting in improved nutrient fermentation and reduced energy losses (32). In line with this hypothesis, Yanza et al. (33) and Yu et al. (10), have shown in a more recent study that certain flavonoid compounds can modulate methanogenesis in the rumen, potentially reducing energy losses associated with methane production. This suggests that Bioflavex may have similar effects on microbial activity in the rumen, contributing to the observed improvements in feed efficiency. Future research should investigate the specific microbial mechanisms involved in the effect of Bioflavex.

Histomorphological measurements of the rumen in lambs, such as papilla length, papilla width, papilla surface area, density, and total papilla surface area, are crucial for assessing the capacity of the rumen for nutrient absorption and digestion (23). These parameters could be valuable for optimizing feeding strategies and improving the growth and health of lambs (34). Therefore, lambs receiving 0.4 and 0.8 g Bioflavex/kg diet dry matter had higher histomorphological parameters in the rumen, including papilla length, papilla width, papilla area and total papilla area, compared to the control group. These results may indicate that Bioflavex can directly or indirectly stimulate the proliferation and differentiation of rumen epithelial cells, leading to stimulation of rumen development and an increase in papilla surface area in lambs. This could be due to improved feed utilization (as observed in the growth performance results) and could thus provide the necessary substrates for rumen tissue growth and development (35). The changes in rumen microbial composition and activity induced by Bioflavex could create a more favorable environment for rumen development [31]. Paniagua et al. [13] reported that the addition of flavonoids to the diet increased the height and width of the rumen papillae in Holstein bulls. In contrast, the addition of Bioflavex had no significant effect on rumen papilla density. This suggests that Bioflavex primarily affected the size and surface area of the papillae present rather than increasing their number.

Lambs fed with Bioflavex showed higher carcass weights at hot and cold than the control group. This is consistent with the improved growth performance observed in these lambs. The lack of significant effects on other carcass characteristics suggests that Bioflavex primarily affects overall body size rather than carcass composition or shape. Furthermore, while no significant differences were found in the relative weights of organs such as liver and spleen, this result is noteworthy. The liver plays a crucial role in nutrient metabolism and its relative weight can be an indicator of metabolic load or efficiency, while the spleen is involved in immune function (36). The lack of change in liver weight suggests that Bioflavex did not impose a significant metabolic burden on the lambs despite the observed improvements in growth performance. The lack of change in relative spleen weight may also indicate that Bioflavex did not significantly alter the immune status of the lambs under the conditions of this study. It is possible that the observed growth improvements were achieved through improved nutrient utilization and/or other physiological mechanisms without significantly affecting the relative weight of the organs. In a previous study, flavonoid administration was found to improve carcass characteristics and organ proportions in lambs (37). Other studies have shown no effect of flavonoid supplementation on carcass characteristics (38, 39). While total carcass weight increased in lambs supplemented with Bioflavex, there was no difference in half carcass weight between dietary treatments. This indicates that Bioflavex did not significantly alter total half carcass size. Lambs fed Bioflavex (0.4 and 0.8 g Bioflavex/kg diet dry matter) had the lowest relative shoulder weight and the highest relative foreshank and breast weights. The highest addition of Bioflavex (0.8 g Bioflavex/kg diet dry matter) resulted in an increase in loin and leg weights. These results may indicate that Bioflavex can influence carcass composition in Awassi lambs. This could be due to the observed changes in the relative weight of carcass parts associated with altered muscle growth and development. Administration of Bioflavex had no effect on the relative weight of internal fat stores, including kidney knob, pericardial and omental fat. In contrast, administration of 0.4 and 0.8 g Bioflavex/kg diet dry matter resulted in a reduction in the thickness of back fat and body wall fat. The reduction in subcutaneous fat thickness is a positive result as it can improve meat quality and consumer acceptance (40).

Color and pH are considered important indicators of lamb meat quality and one of the basic options that give consumers an impression when choosing fresh meat (41). Some studies have shown that lambs fed with polyphenols and flavonoids have no effect on the myoglobin and metmyoglobin content of lamb meat, as the color stability of meat depends on its oxidation and composition (42, 43). However, the main physicochemical properties of the carcass and meat, pH and color (lightness, redness, and yellowness values), were not affected by Bioflavex. Our results are in agreement with those of Liu et al. (44) and Abdallah et al. (45), who observed no pH and color changes in the meat of lambs supplemented with flavonoids. This could indicate a positive result, as maintaining consistent meat quality is crucial for consumer acceptance. However, a slight decrease in the initial and final reddening (redness value) of the carcass walls was observed in the Bioflavex groups compared to the control group. From a practical point of view, even subtle changes in the shade of red can influence consumers’ perceptions and purchasing decisions. In some markets, a bright red color is highly valued and associated with freshness and quality. Despite the lack of statistical significance, this slight decrease in redness is therefore worth considering. It is possible that this change may lead to a perceived decrease in meat quality in certain market segments or consumer groups, potentially impacting market value. Future studies should consider consumers’ sensory evaluations in addition to instrumental color measurements to better understand the practical implications of these subtle color changes.

Cooking loss was lower in lambs fed T3, followed by T2 than T1. This suggests that the meat of lambs fed Bioflavex retained more moisture during cooking, which is desirable for better juiciness and flavor (46). Interestingly, the Bioflavex groups also showed lower water holding capacity. This seems to contradict the lower cooking losses observed. We hypothesize that this discrepancy may be due to changes in protein structure or interactions in muscle tissue. In particular, factors such as oxidation of myofibrillar proteins, changes in protein solubility or changes in the spacing and arrangement of myofibrils could explain these results (47). In the future, researchers should investigate these mechanisms to learn more about how Bioflavex affects the quality of meat. In addition, our results showed that shear force values corresponded with the myofibril fragmentation index, which was lower at 0.4 and 0.8 g Bioflavex. This could indicate lower protein degradation, which is generally associated with improved tenderness in Bioflavex-fed lambs. The lower myofibril fragmentation index (a measure of muscle fiber degradation) in these groups supports this conclusion (48). However, our results were similar to the findings of Orzuna-Orzuna et al. (39), who found that cooking loss and shear force were reduced by plant sources containing flavonoids. The analysis of the texture profile, including hardness, elasticity and cohesion, was not affected by the dietary treatments. However, the lambs fed 0.4 g Bioflavex showed a significantly higher chewing capacity, which may lead to a good consumer perception of meat texture. These results suggest that Bioflavex may have some effects on meat quality parameters in Awassi lambs. Several studies have reported that the addition of flavonoids from the leaves of the Allium plant to the diet improves the tenderness and quality of the meat of lambs (44, 49).

## 5 Conclusion

In conclusion, the lambs fed Bioflavex showed a higher body weight, daily weight gain and RGR compared to the control group, indicating improved nutrient utilization. Histomorphological analysis showed that the addition of Bioflavex increased the length, width, and total surface area of the rumen papillae, indicating stimulated rumen development and possibly improved nutrient uptake. Lambs fed Bioflavex had a higher carcass weights, lower back fat and body wall fat thickness, and lower cooking losses. Bioflavex-fed lambs had lower shear force values and improved chewing ability, indicating improved meat tenderness. Based on the observed improvements in growth, rumen development, and meat quality, particularly with the 0.8 g Bioflavex/kg diet dry matter addition, this level is recommended for optimal performance in Awassi lambs. Overall, the study showed that supplementation with citrus flavonoids can have a positive effect on lamb growth, rumen development and meat quality.

## Data Availability

The original contributions presented in the study are included in the article/supplementary material, further inquiries can be directed to the corresponding author.
